# Supplementary material for: The direct correlation between oxidative stress and LDL-C levels in adults is maintained by the Friedewald and Martin equations, but the methylation levels in the MTHFR and ADRB3 genes differ
Source: PLoS One. 2020 Dec 16;15(12):e0239989. doi: 10.1371/journal.pone.0239989 (PMC7743960; doi:10.1371/journal.pone.0239989)
Supplement: S1 Table — (DOCX) [file pone.0239989.s001.docx]

S1 Table . Concentrations of LDL-C in individuals with high cardiovascular risk based on an estimated arterial pressure of 160 mmHg (5%).

| LDL-C Friedewald | LDL-C Martin | Medication |
| --- | --- | --- |
| 107 | 104 | Captopril |
| 33 | 43 | Hemax, Clopingel, Domperidona |
| 155 | 198 | Valeriane, Tenadren |
| 240 | 237 | Simvastatin, Captopril |
| 82 | 118 | Colchicine, Atenolol, Captopril |
| 94 | 110 | Losartan, Diclofenac |
| 60 | 87 | Losartan, Hydrochlorothiazide |
| 145 | 160 | Enalapril maleate |
| 72 | 85 | Omeprazole, Simvastatin, Metformin |
| 212 | 209 | Losartan |

* p < 0.005; Legend: LDL-C: low-density lipoprotein cholesterol; mean LDL-C F Friedewald = 120 mg/dL; mean LDL-C Martin = 135 mg/dL.
