# Supplementary material for: The direct correlation between oxidative stress and LDL-C levels in adults is maintained by the Friedewald and Martin equations, but the methylation levels in the MTHFR and ADRB3 genes differ
Source: PLoS One. 2020 Dec 16;15(12):e0239989. doi: 10.1371/journal.pone.0239989 (PMC7743960; doi:10.1371/journal.pone.0239989)
Supplement: S2 Table — (DOCX) [file pone.0239989.s002.docx]

S2 Table. Concentrations of LDL-C in individuals with high cardiovascular risk based on an estimated arterial pressure of 140 mmHg (3%).

| LDL-C F mg/dL | LDL-C M mg/dL | Medication |
| --- | --- | --- |
| 107 | 104 | Captopril |
| 131 | 136 | Rivotril, Metformin, Afranil |
| 56 | 94 | Metformin, Omeprazole |
| 240 | 237 | Simvastatin, Captopril |
| 82 | 118 | Colchicine, Atenolol, Captopril |
| 94 | 110 | Losartan, Diclofenac |

* p < 0.005; Legend: LDL-C: low-density lipoprotein cholesterol; F- Friedewald; M- Martin; mean LDL-C F = 118 mg/dL; mean LDL-C M = 133 mg/dL
