# Supplementary figures and images for: The direct correlation between oxidative stress and LDL-C levels in adults is maintained by the Friedewald and Martin equations, but the methylation levels in the MTHFR and ADRB3 genes differ
Source: PLoS One. 2020 Dec 16;15(12):e0239989. doi: 10.1371/journal.pone.0239989 (PMC7743960; doi:10.1371/journal.pone.0239989)

Declaration for experiments involving humans


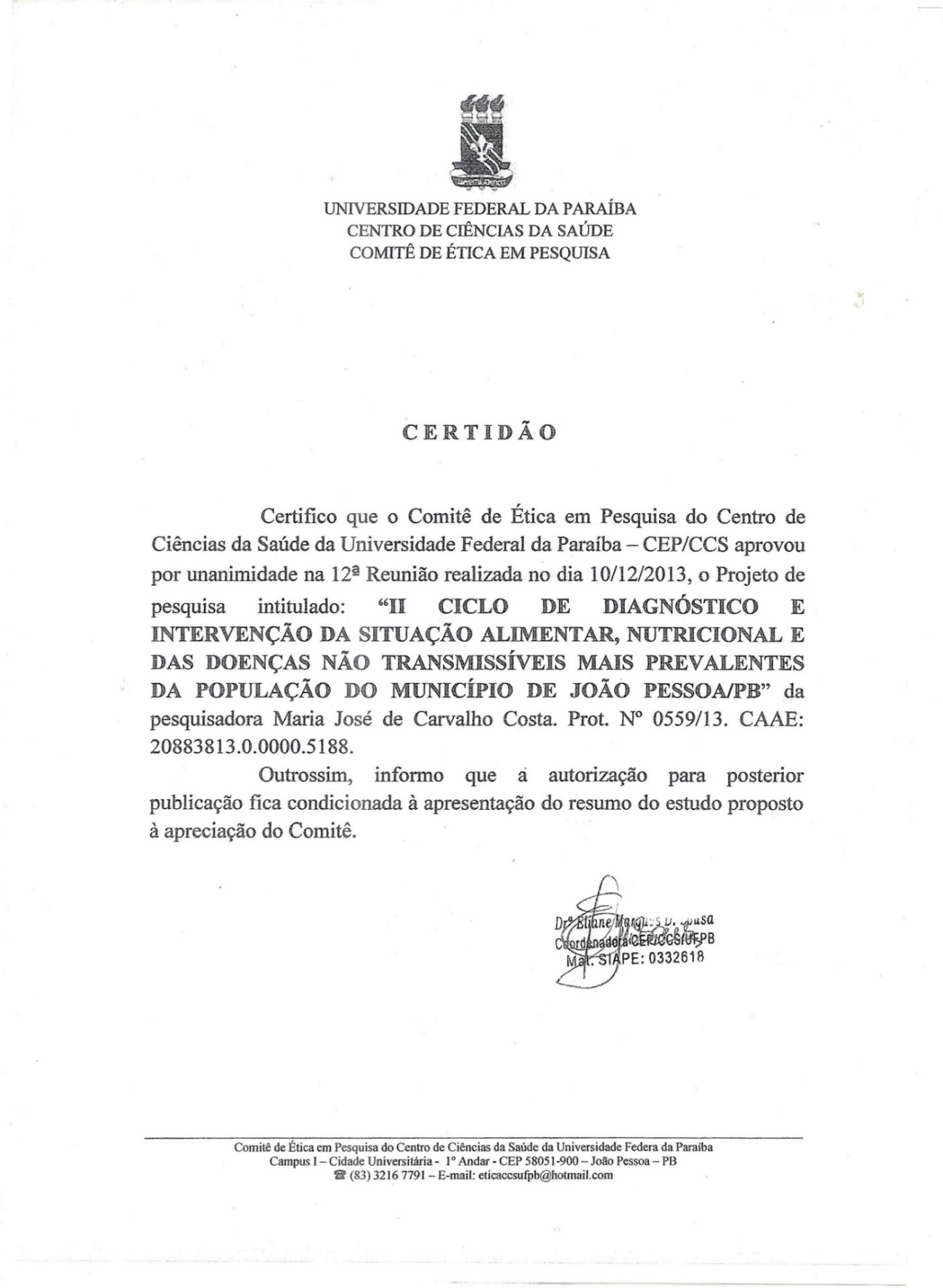

Supplement: S1 File — (DOCX) [file pone.0239989.s003.docx]
