# Supplementary material for: The direct correlation between oxidative stress and LDL-C levels in adults is maintained by the Friedewald and Martin equations, but the methylation levels in the MTHFR and ADRB3 genes differ
Source: PLoS One. 2020 Dec 16;15(12):e0239989. doi: 10.1371/journal.pone.0239989 (PMC7743960; doi:10.1371/journal.pone.0239989)
Supplement: Attachment — Submitted filename: Response to reviewers (1).docx [file pone.0239989.s005.docx]

A rebuttal letter that answers each point raised by the academic editor and reviewer.

Journal Requirements:

When submitting your revision, we need you to address these additional requirements.

1. Please ensure that your manuscript meets PLOS ONE's style requirements, including those for file naming. The PLOS ONE style templates can be found at <https://journals.plos.org/plosone/s/file?id=wjVg/PLOSOne_formatting_sample_main_body.pdf> and <https://journals.plos.org/plosone/s/file?id=ba62/PLOSOne_formatting_sample_title_authors_affiliations.pdf>

2. Our internal editors have evaluated your manuscript and determined that it is within the scope of our 'Primary and Secondary Prevention of Cardiovascular Disease' Call for Papers. This collection of papers is headed by a team of Guest Editors for PLOS ONE and will encompass a diverse range of research articles. Additional information can be found on our announcement page: (<https://collections.plos.org/s/prevention-cardiovascular>). If you would like your manuscript to be considered for this collection, please let us know in your cover letter and we will ensure that your paper is treated as if you were responding to this call. If you would prefer to remove your manuscript from collection consideration, please specify this in the cover letter.

3. Thank you for stating the following in the Acknowledgments Section of your manuscript:

'We thank the funding agencies that made the project possible: Brazilian National Council for
Scientific and Technological Development (Conselho Nacional Desenvolvimento Científico e
Tecnológico - CNPq), Ministry of Health, and Research Support Foundation of the State of
Paraíba (Fundação de Amparo à Pesquisa do Estado da Paraíba - FAPESQ); and the research
collaborators (Graduate Program in Nutritional Sciences, University of Paraíba).'

We note that you have provided funding information that is not currently declared in your Funding Statement. However, funding information should not appear in the Acknowledgments section or other areas of your manuscript. We will only publish funding information present in the Funding Statement section of the online submission form.

Please remove any funding-related text from the manuscript and let us know how you would like to update your Funding Statement. Currently, your Funding Statement reads as follows:

**Answer: Withdrawn.**

'NO - Include this sentence at the end of your statement: The funders had no role in
study design, data collection and analysis, decision to publish, or preparation of the
manuscript.'

4. In your Data Availability statement, you have not specified where the minimal data set underlying the results described in your manuscript can be found. PLOS defines a study's minimal data set as the underlying data used to reach the conclusions drawn in the manuscript and any additional data required to replicate the reported study findings in their entirety. All PLOS journals require that the minimal data set be made fully available. For more information about our data policy, please see <http://journals.plos.org/plosone/s/data-availability>.

Upon re-submitting your revised manuscript, please upload your study’s minimal underlying data set as either Supporting Information files or to a stable, public repository and include the relevant URLs, DOIs, or accession numbers within your revised cover letter. For a list of acceptable repositories, please see <http://journals.plos.org/plosone/s/data-availability#loc-recommended-repositories>. Any potentially identifying patient information must be fully anonymized.

Important: If there are ethical or legal restrictions to sharing your data publicly, please explain these restrictions in detail. Please see our guidelines for more information on what we consider unacceptable restrictions to publicly sharing data: <http://journals.plos.org/plosone/s/data-availability#loc-unacceptable-data-access-restrictions>. Note that it is not acceptable for the authors to be the sole named individuals responsible for ensuring data access.

We will update your Data Availability statement to reflect the information you provide in your cover letter.

**Answer: The database of the present study is present in the public repository, and can be viewed through the link: https://osf.io/t6zn8/?view_only=026188923f00464b8ab252bbc74ef129**

5. PLOS requires an ORCID iD for the corresponding author in Editorial Manager on papers submitted after December 6th, 2016. Please ensure that you have an ORCID iD and that it is validated in Editorial Manager. To do this, go to ‘Update my Information’ (in the upper left-hand corner of the main menu), and click on the Fetch/Validate link next to the ORCID field. This will take you to the ORCID site and allow you to create a new iD or authenticate a pre-existing iD in Editorial Manager. Please see the following video for instructions on linking an ORCID iD to your Editorial Manager account: <https://www.youtube.com/watch?v=_xcclfuvtxQ>

**Answer:** **Jéssica Vicky Bernardo de Oliveira ; ORCID: 0000-0002-4912-6849**

Additional Editor Comments (if provided):

Reviewers' comments:

Reviewer's Responses to Questions

**Comments to the Author**

1. Is the manuscript technically sound, and do the data support the conclusions?

The manuscript must describe a technically sound piece of scientific research with data that supports the conclusions. Experiments must have been conducted rigorously, with appropriate controls, replication, and sample sizes. The conclusions must be drawn appropriately based on the data presented.

Reviewer #1: Partly

2. Has the statistical analysis been performed appropriately and rigorously?

Reviewer #1: N/A

3. Have the authors made all data underlying the findings in their manuscript fully available?

The [PLOS Data policy](http://www.plosone.org/static/policies.action#sharing) requires authors to make all data underlying the findings described in their manuscript fully available without restriction, with rare exception (please refer to the Data Availability Statement in the manuscript PDF file). The data should be provided as part of the manuscript or its supporting information, or deposited to a public repository. For example, in addition to summary statistics, the data points behind means, medians and variance measures should be available. If there are restrictions on publicly sharing data—e.g. participant privacy or use of data from a third party—those must be specified.

Reviewer #1: Yes

4. Is the manuscript presented in an intelligible fashion and written in standard English?

PLOS ONE does not copyedit accepted manuscripts, so the language in submitted articles must be clear, correct, and unambiguous. Any typographical or grammatical errors should be corrected at revision, so please note any specific errors here.

Reviewer #1: Yes

5. Review Comments to the Author

Please use the space provided to explain your answers to the questions above. You may also include additional comments for the author, including concerns about dual publication, research ethics, or publication ethics. (Please upload your review as an attachment if it exceeds 20,000 characters)

Reviewer #1: The paper “The direct correlation between oxidative stress and LDL-C levels in adults is maintained by the Friedewald and Martin equations, but the methylation levels in the MTHFR and ADRB3 genes differ” by Jéssica Vicky Bernardo de Oliveira et al. is of some interest but I have some doubts which are difficult to be showed because the text is without pages and lines number. Therefore, I have showed the words-sentences and some comments have been done.
The number of pages is considering the whole document I received (sorry, but next time I shall not make any referee work on similar situation).

Introduction:

- Pag. 8, the sentence: “The biochemical, anthropometric and consumption variables are justified based on the following description:“ does not seem to be complete.

**Answer: Phrase withdrawn**

Materials and Methods
- Pag. 11, …” Weight and height measurements were carried out in triplicate…” did they occurred at the 3 times of dietary recalls? Please to explain;

**Answer: Weight and height measurements were taken three times in the same day, so that there was no error.**

- Pag. 13, it is suggested: “blood samples were collected at 8:30 a.m. ± 10 min from all individuals to avoid a potential sampling effect.” Please to explain the reason.

**Answer:** **It is known that the gene expression of nucleated blood cells changes ex-vivo shortly after collection [1]. In this sense, even though the DNA suffers less interference from pre-analytical variables, because it has the nitrogen bases more protected in the double helix, with a minimum space for enzymatic attacks [2], a schedule that allows the shortest time between collection was standardized, transport and storage of samples.**

[1] Malentacchi F, Pazzagli M, Simi L, Orlando C, Wyrich R, Gunther K et al. SPIDIA-RNA: second external quality assessment for the pre-analytical phase of blood samples used for RNA based analyses. PLoS One. 2014; 9:e112293.

[2] Ziehler WA, Engelke DR. Probing RNA structure with chemical reagentes and enzymes. Curr Protoc Nucleic Acid Chem 2001; Chapter 6:Unit 6 1.

Results

- Pag. 18, Table 2 I cannot imagine that Homocysteine could be “mol/L”

**Answer: The typo was corrected. µmol / L**

- Pag. 19, Table 3 only dietary cholesterol has been included? See some notes in Portuguese

**Answer: The habitual consumption variables added in model 3 of the statistics were: CHO, PTN, LIP, vitamin A, beta-carotene, vitamin C, vitamin E, vitamin B12, polyunsaturated fats, monounsaturated fats, oleic acid, omega 3, omega 6, saturated fat, cholesterol, trans fats, selenium and folate, however, only the values of the variable (cholesterol) that showed a significant association with LDL-C values were added in Table 3.**

**Table 1 shows the blood cholesterol data.**

**The Portuguese phrase was translated into English.**

- Pag. 20, Table 4 the LPL gene was not included in model 5?

**Answer: It has been added.**

Discussion
- Pag. 21, the following sentence: “The results from the only study found in the literature that compared the LDL-C levels estimated using the two equations in an adult population cannot be compared with the results of the present study because the objectives were different, considering that no comparisons were made between the two equations but, rather, between their results and the direct method” suggests me a question to the authors: Why you did not measure directly the LDL-C or why you did not discuss the two calculated values on the light of papers which have done this comparison? (in Pag. 24 this was considered a limitation of the paper: “Another limitation is the fact that the LDL-C values were not assessed by the direct method”);

**Answer: Although it is important to accurately assess LDL-C through direct measurements, in routine clinical practice, LDL-C levels are generally estimated worldwide using the Friedewald formula [3], when triglyceride levels are below 400 mg / dL. The high cost of analysis of direct measurement methods (such as homogeneous enzyme assay or use of ultracentrifuge) made the use in the present population-based study unfeasible.**

3.	Catapano AL, Graham I, de Backer G, Wiklund O, Chapman MJ, Drexel H, et al. 2016 ESC/EAS Guidelines for the Management of Dyslipidaemias. Rev Española Cardiol. 2017;70(2):115.

# Zabłocka-Słowińska, k. Oxidative stress in lung cancer patients is associated with altered serum markers of lipid metabolism. PLOS ONE, 2019. Doi: [10.1371/journal.pone.0215246](https://dx.doi.org/10.1371%2Fjournal.pone.0215246)

# The results of the study by Lee et al. (14) are presented according to the agreement with the direct method, which makes the comparison unfeasible. Lee et al. (14) observed that the Martin equation exhibited significantly higher overall agreement with the classifications of the NCEP-ATP III guidelines, than the Friedewald equation, as already described in the present article.

14. Lee J, Jang S, Son H. Validation of the Martin Method for Estimating Low-Density Lipoprotein Cholesterol Levels in Korean Adults: Findings from the Korea National Health and Nutrition Examination Survey, 2009-2011. Passi AG, editor. PLoS One. 2016;11(1):1–14.

# - Pag. 21, the following sentence; “MDA was the only variable that positively influenced LDL-C concentrations, calculated based on the Friedewald (36) and Martin et al. “ but are you sure that MDA influence LDL-C and not vice versa?

# In the study by Yang et al. [23] it was demonstrated that the impairment of the integrity and survival of human coronary artery endothelial cells (HCAECs) induced by oxidized LDL is mediated by the MDA epitope and involves the methylation of the prosurvival fibroblast growth factor 2 (FGF2) promoter and the negative regulation of intracellular FGF2. Signaling pathway through which MDA can mediate LDL-C oxidation.

23. Yang TC, Chen YJ, Chang SF, Chen CH, Chang PY, Lu SC. Malondialdehyde mediates oxidized LDL-induced coronary toxicity through the Akt-FGF2 pathway via DNA methylation. J Biomed Sci. 2014;21(1):1–12.

# According to the reading of the scientific community, we observed that there is no consensus regarding the questioned influence. However, in the present study we identified that the MDA was positively related to the LDL-C values.

- Pag. 22, there are different sentences suggesting a relationship between DNA methylation of some genes and the levels of LDL-C or some data like obesity, fat storages etc. Sorry, I am not an expert of the topic, but I want pose two questions: i) the epigenetic effects would be at tissue level, thus can you measure methylation at blood level? ii) methylation can have different effects according to the gene site in which occurred, can you consider that?

**Answer: We fully understand your questioning, which was justified in our published article.**

# Lima, R.P.A.; NASCIMENTO, R.A.F. Effect of a diet containing folate and hazelnut oil capsule on the methylation level of the *ADRB3* gene, lipid profile and oxidative stress in overweight or obese women, Clinical Epigenetics, 2017. DOI [10.1186/s13148-017-0407-6](https://dx.doi.org/10.1186%2Fs13148-017-0407-6).

**The blood was chosen for the analysis as it is a metabolically active tissue, with an important role in the adverse inflammatory and vascular consequences of adiposity, and is widely used for clinical diagnostic purposes.**

- Pag. 23, there is a sentence: “the population presented high MDA values, these values likely affect the lipid profile, increasing the LDL-C concentrations in the analysed models in the study population, with 75% to 67% of the LDL-C concentrations considered adequate, according to the two equations used.” But, again, MDA is more likely affected by lipid profile and perhaps by micro-inflammatory conditions which occur when there is obesity and high LDL-C;

**Answers: As suggested, changes were made to the text.**

**Malondialdehyde (MDA) is a final product of oxidative decomposition initiated by radical polyunsaturated fatty acids; therefore, it is often used as a biomarker of oxidative stress (23).**

23. Yang TC, Chen YJ, Chang SF, Chen CH, Chang PY, Lu SC. Malondialdehyde mediates oxidized LDL-induced coronary toxicity through the Akt-FGF2 pathway via DNA methylation. J Biomed Sci. 2014;21(1):1–12.

[59] Lopes-Virella MF, Hunt KJ, Baker NL, Virella G, Moritz T. The levels of MDA-LDL in circulating immune complexes predict myocardial infarction in the VADT study. Atherosclerosis. 2012;224(2):526–31.

- Pag. 23, “…several studies have shown that MDA and LDL-C values decrease in animal models with interventions based on the intake of different foods (21, 22, 27).” First of all it would be useful to better explain which foods are decreasing the LDL-C, moreover I am asking why very few or no dietary data of the experiment are been discussed;

**Answers:** Regarding the relationship between MDA and LDL-C, several studies have shown that MDA and LDL-C values decrease in animal models with interventions based on the intake of different foods**: Garlic (*Allium sativum* L.) polysaccharide (21), Chicory (*Cichorium intybus* L.) polysaccharides (22), *Morechella esculenta* polysaccharide (27).**

As for dietary consumption, the authors were concerned about the extent of the discussion. If necessary, we add.

21. Wang Y, Guan M, Zhao X, Li X. Effects of garlic polysaccharide on alcoholic liver fibrosis and intestinal microflora in mice. Pharm Biol. 2018;56(1):325–32.

22. Wu Y, Zhou F, Jiang H, Wang Z, Hua C, Zhang Y. Chicory (Cichorium intybus L.) polysaccharides attenuate high-fat diet induced non-alcoholic fatty liver disease via AMPK activation. Int J Biol Macromol. 2018;118:886–95.

27. Dong Y, Qi Y, Liu M, Song X, Zhang C, Jiao X, et al. Antioxidant, anti-hyperlipidemia Land hepatic protection of enzyme-assisted Morehella esculenta polysaccharide. Int J Biol Macromol. 2018;120(2018):1490–9.

- Pag. 24, in fact, in the Pag. 24, some sentences are concerned with the above topic: “…here was a positive influence of age, habitual cholesterol intake and WC on LDL-C levels.” Then “…even when the total fat intake was within the reference range (29%) (70) and the habitual cholesterol intake was 243.51 mg, an influence of this intake on LDL-C levels was identified. The explanation for this discrepancy may involve the type of fat consumed.” These topics are very important for a better explanation of the results and therefore the authors are invited to show the results of the dietary survey and to discuss them

**Answer: We added to the discussion as requested, however, we did not add the tables due to the length of the article, but if it is convenient for the reviewers, we will add it.**

Another explanation for this discrepancy may involve the type of fat consumed, since, in the case of the present study, the consumption of saturated fatty acids was 12%, exceeding the recommendation according to the National Institute of Heart, Lung and Blood: Detection, assessment and treatment of high blood cholesterol levels in adults (adult treatment panel III), final report, US Department of Health and Human Services, NIH Publication No. 02-5215, Bethesda, Maryland, September 2002.

Conclusions

Must be changed according to the suggested improvements of the discussion.

6. PLOS authors have the option to publish the peer review history of their article ([what does this mean?](https://journals.plos.org/plosone/s/editorial-and-peer-review-process#loc-peer-review-history)). If published, this will include your full peer review and any attached files.


If you choose “no”, your identity will remain anonymous but your review may still be made public.


**Do you want your identity to be public for this peer review?** For information about this choice, including consent withdrawal, please see our [Privacy Policy](https://www.plos.org/privacy-policy).

Reviewer #1: No

[NOTE: If reviewer comments were submitted as an attachment file, they will be attached to this email and accessible via the submission site. Please log into your account, locate the manuscript record, and check for the action link "View Attachments". If this link does not appear, there are no attachment files to be viewed.]


While revising your submission, please upload your figure files to the Preflight Analysis and Conversion Engine (PACE) digital diagnostic tool, <https://pacev2.apexcovantage.com/>. PACE helps ensure that figures meet PLOS requirements. To use PACE, you must first register as a user. Registration is free. Then, login and navigate to the UPLOAD tab, where you will find detailed instructions on how to use the tool. If you encounter any issues or have any questions when using PACE, please email us at [figures@plos.org](mailto:figures@plos.org). Please note that Supporting Information files do not need this step.
